# Ultrasound Entropy Imaging of Nonalcoholic Fatty Liver Disease: Association with Metabolic Syndrome

**DOI:** 10.3390/e20120893

**Published:** 2018-11-22

**Authors:** Ying-Hsiu Lin, Yin-Yin Liao, Chih-Kuang Yeh, Kuen-Cheh Yang, Po-Hsiang Tsui

**Affiliations:** 1 Department of Medical Imaging and Radiological Sciences, College of Medicine, Chang Gung University, Taoyuan 33302, Taiwan; 2 Department of Biomedical Engineering, Hungkuang University, Taichung 43302, Taiwan; 3 Department of Biomedical Engineering and Environmental Sciences, National Tsing Hua University, Hsinchu 30013, Taiwan; 4 Department of Family Medicine, National Taiwan University Hospital, Beihu Branch, Taipei 10800, Taiwan; 5 Health Science & Wellness Center, National Taiwan University, Taipei 10617, Taiwan; 6 Department of Medical Imaging and Intervention, Chang Gung Memorial Hospital at Linkou, Taoyuan 33305, Taiwan; 7 Medical Imaging Research Center, Institute for Radiological Research, Chang Gung University and Chang Gung Memorial Hospital at Linkou, Taoyuan 33302, Taiwan

**Keywords:** ultrasound, hepatic steatosis, Shannon entropy, fatty liver, metabolic syndrome

## Abstract

Nonalcoholic fatty liver disease (NAFLD) is the leading cause of advanced liver diseases. Fat accumulation in the liver changes the hepatic microstructure and the corresponding statistics of ultrasound backscattered signals. Acoustic structure quantification (ASQ) is a typical model-based method for analyzing backscattered statistics. Shannon entropy, initially proposed in information theory, has been demonstrated as a more flexible solution for imaging and describing backscattered statistics without considering data distribution. NAFLD is a hepatic manifestation of metabolic syndrome (MetS). Therefore, we investigated the association between ultrasound entropy imaging of NAFLD and MetS for comparison with that obtained from ASQ. A total of 394 participants were recruited to undergo physical examinations and blood tests to diagnose MetS. Then, abdominal ultrasound screening of the liver was performed to calculate the ultrasonographic fatty liver indicator (US-FLI) as a measure of NAFLD severity. The ASQ analysis and ultrasound entropy parametric imaging were further constructed using the raw image data to calculate the focal disturbance (FD) ratio and entropy value, respectively. Tertiles were used to split the data of the FD ratio and entropy into three groups for statistical analysis. The correlation coefficient *r*, probability value *p*, and odds ratio (OR) were calculated. With an increase in the US-FLI, the entropy value increased (*r* = 0.713; *p* < 0.0001) and the FD ratio decreased (*r* = –0.630; *p* < 0.0001). In addition, the entropy value and FD ratio correlated with metabolic indices (*p* < 0.0001). After adjustment for confounding factors, entropy imaging (OR = 7.91, 95% confidence interval (CI): 0.96–65.18 for the second tertile; OR = 20.47, 95% CI: 2.48–168.67 for the third tertile; *p* = 0.0021) still provided a more significant link to the risk of MetS than did the FD ratio obtained from ASQ (OR = 0.55, 95% CI: 0.27–1.14 for the second tertile; OR = 0.42, 95% CI: 0.15–1.17 for the third tertile; *p* = 0.13). Thus, ultrasound entropy imaging can provide information on hepatic steatosis. In particular, ultrasound entropy imaging can describe the risk of MetS for individuals with NAFLD and is superior to the conventional ASQ technique.

## 1. Introduction

Nonalcoholic fatty liver disease (NAFLD) is characterized by excess and abnormal intracellular accumulation of triglycerides in hepatocytes. Histologically, NAFLD refers to macrovesicular steatosis and is the leading cause of nonalcoholic steatohepatitis, fibrosis, cirrhosis, and hepatocellular carcinoma [1,2]. Therefore, NAFLD may be considered a critical health problem, and its early detection, follow-up, and management can help arrest the progression of advanced liver diseases [3,4].

Currently, liver biopsy is the gold standard for diagnosing NAFLD [5]. However, liver biopsy is an invasive procedure and can lead to serious complications (e.g., bleeding), and its diagnosis may be inconsistent between pathologists [6,7]. Moreover, sampling errors limit the use of liver biopsy in clinical practice. Additionally, most patients with NAFLD have no significant clinical symptoms, and performing liver biopsies on such patients is ethically controversial. To resolve this dilemma, noninvasive imaging modalities such as ultrasound, computed tomography, magnetic resonance imaging, and magnetic resonance spectroscopy (MRS) are commonly used for the assessment of hepatic steatosis [8]. Ultrasound imaging provides several advantages, including ease of routine examination, cost-effectiveness, portability, and nonionizing imaging principles, and thus it is currently the first-line modality for assessing hepatic steatosis and evaluating NAFLD.

Ultrasound performs well in detecting moderate to severe hepatic steatosis [9,10]. However, its diagnostic accuracy for detecting mild hepatic steatosis is limited. Furthermore, qualitative descriptions, operator experience, and interobserver and intraobserver variability degrade the sonographic assessment of fatty liver [11,12]. Quantitative analysis of ultrasound images may provide additional clues to improve the diagnosis of mild NAFLD. Essentially, liver parenchyma can be modeled as a scattering medium consisting of numerous acoustic scatterers [13,14] that interact with the incident wave to form ultrasound backscattered signals. Different scatterer properties result in different waveforms of backscattered signals, and thus the corresponding statistical properties may depend on information associated with changes in liver microstructures [13].

Considering the randomness of ultrasound backscattering, statistical distributions are widely used to model backscattered statistics for tissue characterization [15]. Nakagami [16,17] and homodyned-K distributions [18] have been applied to model ultrasound backscattered statistics for the assessment of hepatic steatosis. However, acoustic structure quantification (ASQ) based on Chi-squared testing of backscattered envelopes is the only technique that has been commercialized in ultrasound scanners (Toshiba machine) by using the concept of statistical distribution. Initially, ASQ was developed to quantify the difference between backscattered statistics and Rayleigh distribution [19]. ASQ has been validated as having high performance in evaluating NAFLD because fat accumulation in the liver tends to make the statistics of backscattered data follow the Rayleigh distribution [20,21,22,23,24].

When using ASQ or model-based methods to characterize tissue, the data used to estimate the parameters must conform to the used statistical distribution [25,26]. This requirement may not always be satisfied, because adjusting the settings in an ultrasound system or using nonlinear signal-processing approaches (e.g., logarithmic compression) may alter the statistical distribution of raw data. This limitation has motivated researchers to consider non-model-based statistical approaches. Among all possible approaches, Shannon entropy—an estimate of signal uncertainty and complexity proposed in information theory [27]—has the highest potential and flexibility for analyzing ultrasound backscattering. Hughes first proposed using information (Shannon) entropy to analyze ultrasound signals, indicating that entropy can be used to quantitatively depict changes in the microstructures of scattering media [28,29]. In particular, one report demonstrated that information entropy can describe ultrasound backscattered statistics without considering the statistical properties of ultrasound data [30]. Recent studies have further indicated that entropy parametric imaging enables visualization and characterization of hepatic steatosis, thereby making it possible to implement non-model-based structure quantification of NAFLD [31,32,33].

While non-model-based entropy imaging plays an increasingly key role in physically describing changes in the microstructures of fatty liver, its meanings require further biological explanation. The establishment and validation of ultrasound entropy imaging to characterize hepatic steatosis are based on the association of entropy value with hepatic histological changes [33]. However, NAFLD is not only a change in liver microstructures caused by fat accumulation but also strongly related to obesity, hypertension, type 2 diabetes mellitus, and dyslipidemia, all of which are metabolic abnormalities and can be considered hepatic manifestations of metabolic syndrome (MetS) [34,35]. MetS is typically caused by insulin resistance, and although glucose clamp is the gold standard for quantifying insulin resistance, it is a complex procedure that is unsuitable for routine use. For this reason, Matthews et al. developed the homeostatic model assessment for insulin resistance (HOMA-IR) index, which is calculated using fasting insulin and blood glucose for a general evaluation of MetS [36]. The HOMA-IR index correlates with the conventional ultrasound B-scan image features of hepatic steatosis [37,38], implying that ultrasound imaging can depict metabolic information. Therefore, we explored the relationship between MetS and quantitative ultrasound analysis of NAFLD by using entropy imaging.

This study had two objectives: (i) investigating the association of ultrasound entropy imaging of NAFLD with MetS to endow entropy images with new biological insights, and (ii) comparing the performance of entropy imaging in predicting the risks of suffering from MetS with that of conventional ASQ to determine whether non-model-based approaches are at all superior for evaluating MetS. The results showed that ultrasound entropy imaging performed well in describing the metabolic behavior of patients with NAFLD. Moreover, ultrasound entropy imaging was superior to ASQ in risk evaluation for MetS.

## 2. Materials and Methods

### 2.1. Subjects

This study was conducted following approval by the Institutional Review Board of National Taiwan University Hospital. All participants were asked to complete standardized questionnaires and provided informed consent. Participants with the following conditions were excluded: excessive alcohol intake (>20 g/day for women and >30 g/day for men) and chronic liver disease (chronic hepatitis, autoimmune, drug-induced, vascular, or inherited hemochromatosis or Wilson disease). A total of 394 patients were recruited.

### 2.2. Anthropometric Indices and Biochemical Analyses

Routine physical examinations and blood tests were conducted for each participant. Body mass index (BMI) was calculated as weight divided by height squared. Waist circumference (WC) was measured at the middle between the costal margin and iliac crest. Systolic blood pressure (SBP) and diastolic blood pressure (DBP) were recorded. Fasting plasma glucose (FPG), total cholesterol (TCH), triglycerides (TG), high-density lipoprotein (HDL-C), low-density lipoprotein (LDL-C), aspartate aminotransferase (AST), alanine aminotransferase (ALT), and insulin were measured after 8 hours of overnight fasting. Using FPG and insulin, the HOMA-IR index was calculated to examine insulin resistance [36].

### 2.3. Diagnosis of MetS

Data obtained from anthropometric and blood examinations were further used to identify MetS. According to the modified National Cholesterol Education Program Adult Treatment Panel III Criteria (NCEP-ATP III), MetS (for the Taiwanese population) is diagnosed when at least three of the following criteria are satisfied [39]: (i) WC ≥ 90 cm in men and ≥ 80 cm in women; (ii) SBP ≥ 130 mmHg or DBP ≥ 85 mmHg or use medication for hypertension; (iii) hyperglycemia (FPG ≥ 100 mg/dL) or the use of medication for diabetes; (iv) hypertriglyceridemia (TG ≥ 150 mg/dL) or use of medication for hyperlipidemia; and (v) low HDL-C (≤40 mg/dL in men and ≤50 mg/dL in women).

### 2.4. Ultrasound Examinations for NAFLD Evaluation

After blood withdrawal, standard abdominal ultrasound screening of the liver was performed immediately by three physicians, each with more than 20 years’ experience. A clinical ultrasound scanner (Model 3000; Terason, Burlington, MA, USA) equipped with a convex transducer (Model 5C2A; Terason) of 3 MHz was used; the transducer had 128 elements and the pulse length of the incident wave was approximately 2.3 mm. For each participant, the ultrasonographic fatty liver indicator (US-FLI) was used as a semiquantitative measure of severity of NAFLD [40]. Specifically, the US-FLI was calculated using the following criteria: (i) presence of liver/kidney contrast graded as mild/moderate (score 2) or severe (score 3); (ii) presence (score 1 each) or absence (score 0 each) of posterior attenuation of ultrasound beam, vessel blurring, difficult visualization of the gallbladder wall, difficult visualization of the diaphragm, and areas of focal sparing. NAFLD was diagnosed if the score ≥2 [40].

### 2.5. Quantitative Analysis using ASQ and Entropy Imaging

Except for the standard abdominal scans, all physicians followed the same protocols and system settings for data acquisition and quantitative analysis. For each patient, the same scanner was used to scan the liver through the subcostal scanning approach. It has been shown that a signal-to-noise ratio (SNR) > 11 dB allows reliable descriptions of ultrasound backscattered statistics [41]. For this consideration, the system gain index was set at 6, corresponding to a SNR of approximately 30 dB, which was obtained from the calibrations in the previous study [42]. Such a high SNR implies that no significant noise components exist in the backscattered signals, ensuring the quality of parameter estimation in the ASQ analysis. The imaging depth was 16 cm and the focal zone corresponded to the central part of the liver to reduce the effect of beam diffraction. Raw image data consisting of 128 scan lines of backscattered radio frequency signals at a sampling rate of 30 MHz were obtained using the software kit provided by Terason. The envelope image of each raw image raw datum was constructed by taking the absolute value of the Hilbert transform of each scan line. The grayscale B-mode image was formed based on the logarithm-compressed envelope data at a dynamic range of 40 dB.

In ASQ, the Chi-squared test is used to evaluate the difference between the sample and the population data. The following equation is used to define parameter $C_{m}^{2}$ [19]:

(1) $$\;C_{m}^{2}=\frac{\sigma_{m}^{2}}{\sigma_{R}^{2}\left(\mu_{m}\right)}=\left[\frac{\pi }{4-\pi }\right]\frac{\sigma_{m}^{2}}{\mu_{m}^{2}}\;$$

where $\mu_{m}$ and $\sigma_{m}^{2}$ are the average and variance of the measured backscattered envelopes, respectively. The value of $\sigma_{R}^{2}\left(\mu_{m}\right)$ indicates the variance of the Rayleigh-distributed data estimated using $\mu_{m}$. In this study, the sliding window technique was used to obtain a $C_{m}^{2}$ parametric map. In brief, a window was created to move across the entire envelope image in steps representing the number of pixels corresponding to the window overlap ratio (WOR); during this process, local parameters were successively estimated using local envelope data within the window so that a parametric map could eventually be constructed. The window side length (WSL) was three times the pulse length, which is an appropriate size for stably estimating ultrasound statistical parameters [17]. The WOR was 50% to provide a tradeoff between the parametric image resolution and computational time [43]. A region of interest (ROI) manually outlined on the B-mode image of the liver parenchyma was used for analysis of the $C_{m}^{2}$ parametric map. Some basic criteria suggested previously were used for determining the ROI [17]: (i) visible blood vessels were excluded in the ROI to reduce the bias of characterizing liver parenchyma. The size of the ROI was set 3 × 3 cm^2^; (ii) the ROI was located at the focal zone, reducing the effects of attenuation and diffraction on the backscattered signals.

Referring to a previous study [44], the histogram of $C_{m}^{2}$ in the ROI revealed a narrow distribution when the tissue was homogeneous. A relatively broad distribution represented either diffusely inhomogeneous (consisting of microstructures) or focally inhomogeneous (consisting of macrostructures such as vessels) tissue. To eliminate macrostructural information, sliding window processing of the envelope image was performed again (generating a second $C_{m}^{2}$ map denoted as *r*$C_{m}^{2}$ map), where local data in the window were excluded if the amplitude > (*μ* + *ασ*) (*μ*: mean value of envelope data in the ROI, *σ*: standard deviation of envelope data in the ROI, and *α*: a removal coefficient). For each pixel location in the ROI, if the ratio of $C_{m}^{2}$/*r*$C_{m}^{2}$ was lower than the threshold *k*, $C_{m}^{2}$ was considered to exhibit no significant changes after rejecting the outliers of the envelope signals. In this condition, $C_{m}^{2}$ was assigned to the pixel location. If $C_{m}^{2}$/*r*$C_{m}^{2}$ was greater than *k*, *r*$C_{m}^{2}$ was used. Finally, the values of $C_{m}^{2}$ and *r*$C_{m}^{2}$ in the ROI were separated to construct two histograms to represent microstructure (diffuse inhomogeneity or homogeneity) and macrostructure (focal inhomogeneity) curves. The focal disturbance (FD) ratio was defined as the ratio of the area under the curve for $C_{m}^{2}$ and *r*$C_{m}^{2}$ histograms, expressed as

(2) $$\;FD-ratio=\frac{AUC\left(rC_{m}^{2}\;histogram\right)}{AUC\left(C_{m}^{2}\;histogram\right)}\;$$

**Figure 1:**
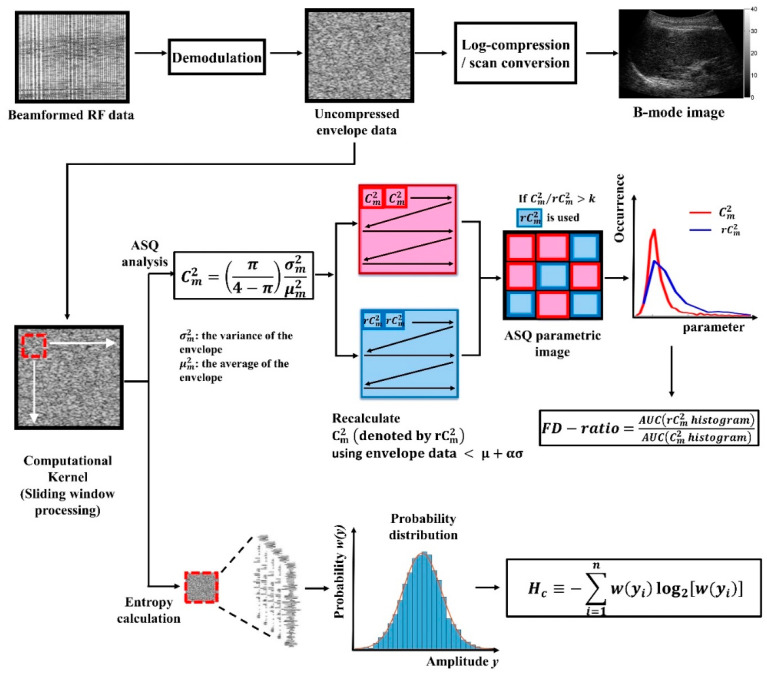
Computational flowchart for ultrasound acoustic structure quantification (ASQ) and entropy estimations.

When the resolution cell of the transducer contains a large number of randomly distributed scatterers, the statistical distribution of ultrasound backscattered envelopes exhibits the Rayleigh distribution [13,14,15]. This condition represents that no macrostructures exist in the tissue to generate information of *r*$C_{m}^{2}$, and thus the FD ratio is theoretically equal to zero. On the contrary, the FD ratio increases with the degree of deviation from Rayleigh statistics [44]. Please note that the removal coefficient α = 7 [19] and the threshold *k* = 1.2 [44] were suggested previously but could be empirically determined [19,45]. Because the initial equipment (Toshiba system with software package) was unavailable in this study, we fine-tuned the parameters for the used Terason system. The values of α and *k* were set at 3 and 1.1, respectively. The algorithmic scheme of estimating the FD ratio is illustrated in Figure 1.

The algorithm for ultrasound entropy imaging is also based on the sliding window technique to process the envelope image and is illustrated in Figure 1. Because the acquired ultrasound backscattered signals digitalized by the imaging system belong to discrete signals, the Shannon entropy of a discrete random variable *Y* with possible values {*y*_1_, *y*_2_, …., *y*_n_} (i.e., the envelope data points included within the sliding window) was calculated using the following discrete form:

(3) $$H_{c}\equiv -\overset{n}{\underset{i=1}{\sum }}w\left(y_{i}\right)\log_{2}\left[w(y_{i})\right]\;$$

where *w*(∙) represents the function of probability distribution. In this study, the statistical histogram of the data (bins = 200) was used as an alternative *w*(∙) for estimation [31,32]. To compare the results of entropy with those of ASQ, ultrasound entropy imaging was constructed using the same WSL (6.9 mm) and WOR (50%). The ROI used in the ASQ analysis was directly applied to the entropy parametric image to calculate the average entropy value.

### 2.6. Statistical Analysis

The Kolmogorov-Smirnov, Anderson-Darling, Cramer-Von Mises, and Shapiro-Wilk tests of the data (the US-FLI, FD ratio, and entropy) were used for normality testing. Tertiles were used to split the data of the FD ratio and entropy into three groups. For each group, the categorical data were presented as percentages and the continuous variables were expressed as mean ± standard deviation. Initially, the interrelationships between the US-FLI, FD ratio, and entropy value were plotted to calculate the Pearson correlation coefficient *r* and probability value *p*. Then, the categorical data were analyzed using the Chi-squared test. The continuous variables in each group were compared using analyses of variance. The Cochran-Armitage trend test was conducted to test for trends in the anthropometric and metabolic factors by using tertiles of the FD ratio and entropy value. The associations of the FD ratio and entropy value with MetS were assessed using a multiple logistic regression model adjusted for age, sex, alcohol consumption, smoking, betel nut chewing, hours of exercise per week, menopause status (women only), BMI, and HOMA-IR. To further compare the abilities of the FD ratio and entropy in predicting the risk of suffering from MetS, the odds ratio (OR) and 95% confidence interval (CI) were calculated. The significant difference was set at *p* < 0.05. All statistical analyses were conducted using SAS version 9.3 (SAS Inc., Cary, NC, USA).

## 3. Results

### 3.1. Baseline Characteristics of the Participants

**Figure 2:**
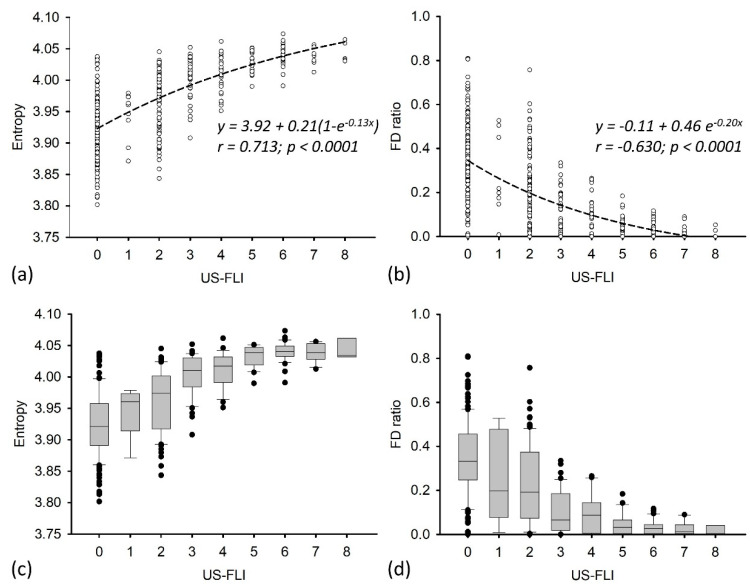
(**a**) and (**b**) Dot plots of entropy value (**left**)/FD (focal disturbance) ratio (**right**) corresponding to each US-FLI. (**c**) and (**d**) Box plots of entropy value (**left**)/FD ratio (**right**) corresponding to each US-FLI.

The baseline characteristics of the participants are shown in Table 1. A total of 394 participants was recruited, comprising 151 (38.3%) men and 243 (61.7%) women (mean age: 40.5 ± 11.3 years). According to information obtained from questionnaires, anthropometric examinations, blood tests, and ultrasound evaluations of NAFLD, the overall prevalence of MetS was 19.3% and the US-FLI, FD ratio, and entropy value of the participants were 2.22 ± 2.25, 0.96 ± 0.44, and 3.99 ± 0.06, respectively. To observe how the statistical properties of backscattered signal varied with the severity of NAFLD, dot and box plots of the FD ratio and entropy value corresponding to each US-FLI were plotted (Figure 2). Based on observations of the data, exponential increasing and decreasing functions were used for fitting dot plots of the entropy and FD ratio, respectively. With an increase in the US-FLI, the FD ratio decreased (*r* = –0.630; *p* < 0.0001) and the entropy value monotonically increased (*r* = 0.713; *p* < 0.0001). Box plots further identified outliers for the entropy and FD ratio. Some outliers were found to exist in the data distributions of the entropy and FD ratio. This is acceptable and reasonable, especially for a large amount of biodata (total n = 394). The US-FLI underestimating the extent of NAFLD [16] is another possible reason for the outliers of entropy and FD ratio. On the other hand, the normality tests based on four kinds of methods (as described in Section 2.6) indicated that the data of the US-FLI, FD ratio, and entropy did not follow the normal distribution (*p* < 0.0001). However, the data distribution does not affect the subsequent analysis (using the OR to evaluate the risk of MetS) because the OR estimations were based on the tertiles of entropy and FD ratio.

### 3.2. Characteristics of Participants in Different Tertiles

Table 2 shows the characteristics of participants in different tertiles of the FD ratio and entropy. No significant difference in age was found (*p* = 0.2602). The percentage in men decreased with an increase in the FD ratio (*p* < 0.0001). Compared with the patients in higher tertiles, those in lower tertiles (lower FD ratios) exhibited lower HDL-C (*p* < 0.0001) and higher WC, BMI, body fat, SBP, DBP, FPG, TCH, TG, LDL-C, MetS, insulin, HOMA-IR, and abnormal liver function (*p* = 0.0353 for TCH; *p* = 0.0022 for LDL-C; *p* < 0.0001 for the others). Similar results were found in the tertiles of entropy. With an increase in entropy (from lower to higher tertiles), WC, BMI, body fat, SBP, DBP, FPG, TCH, TG, LDL-C, MetS, insulin, HOMA-IR, and abnormal liver function increased (all *p* < 0.0001), and HDL-C decreased (*p* < 0.0001). These results revealed that the ultrasound entropy value and FD ratio correlate with MetS.

### 3.3. The Risks of Metabolic Syndrome in Different Tertiles for the FD Ratio and the Entropy Value

The risk of metabolic syndrome in each tertile are compared in Table 3. For the FD ratio, the second tertile (lower FD ratios) exhibited a higher risk of MetS (OR = 0.48; 95% CI: 0.26–0.89) than did the third tertile (OR = 0.04; 95% CI: 0.01–0.14) after use of model 1 adjusted for age, sex, smoking, alcohol consumption, betel nut chewing, hours of exercise per week, and menopause status (*p* < 0.0001). Following further adjustment for BMI (model 2), the ORs in the second and third tertiles were 0.59 (95% CI: 0.30–1.18) and 0.41 (95% CI: 0.16–1.05), respectively (*p* = 0.1144). After use of HOMA-IR to further adjust the OR (model 3), the ORs in the second and third tertile were 0.55 (95% CI: 0.27–1.14) and 0.42 (95% CI: 0.15–1.17), respectively (*p* = 0.13). Notably, entropy improved the performance of predicting the risk of MetS. Through use of model 1, the OR of entropy in the third tertile (85.57; 95% CI: 11.25–650.56) was larger than that in the second tertile (51.29; 95% CI: 2.76–164.43) (*p* < 0.0001). After adjustment using model 2, the OR of entropy in the third tertile (26.84; 95% CI: 3.34–215.4) was higher than that in the second tertile (10.27; 95% CI: 1.29–82.14) (*p* = 0.0007), as in model 3 (OR = 7.91, 95% CI: 0.96–65.18 for the second tertile; OR = 20.47, 95% CI: 2.48–168.67 for the third tertile; *p* = 0.0021). The results indicated that non-model-based entropy provides a stronger link to biologically metabolic information than does conventional ASQ.

## 4. Discussion

### 4.1. Significance of This Study

With the development and commercialization of ultrasound statistical models and parametric imaging, physicians gradually have a new choice for diagnosing NAFLD. As stated in the Introduction, more than one statistical distribution can be used to assess hepatic steatosis, and the ASQ technique has the clinical benefit of using the model-based method to analyze the statistical properties of backscattered signals from fatty liver. The best statistical distribution for modeling the backscattered statistics of NAFLD is yet to be determined. However, ultrasound entropy imaging based on information theory is more adaptive to various signal characteristics because the calculation of entropy does not need to consider the statistical properties of the signal itself. Therefore, when viewing entropy imaging as a new approach for NAFLD diagnosis, it is necessary to not only perform pathological validations but also to explore the metabolic meanings of entropy. Studies have confirmed that the value of ultrasonic entropy is closely related to the pathological changes of hepatic steatosis [32,33]. However, the present study expands our understanding and domain knowledge of ultrasound entropy imaging; we demonstrated that ultrasound entropy imaging can describe the risk of MetS for those with NAFLD and is superior to the conventional ASQ technique.

### 4.2. Effects of NAFLD on FD Ratio and Entropy

The US-FLI was used as a semiquantitative measure of NAFLD in this study. Our results showed that both the FD ratio obtained from ASQ analysis and the entropy value of ultrasound entropy imaging correlated with the US-FLI, indicating that these two parameters vary with the progress of NAFLD because macrovesicular steatosis is the major pathological change of NAFLD. Macrovesicular steatosis refers to the presence of a single large fat droplet in a hepatocyte that pushes the nucleus to the periphery. In this scenario, the number of acoustic scatterers (fat droplets) increases equivalently in the scattering medium (liver parenchyma), and the enhancement of constructive wave interference results in changes in the waveforms of the backscattered signals, making the corresponding backscattered statistics vary from pre-Rayleigh (backscattered statistics for healthy livers in practice) to Rayleigh distribution (hepatic steatosis) [16,17,21]. This explains why the FD ratio ASQ parameter monotonically decreases with an increase in the degree of hepatic steatosis. Concurrently, the effect of constructive wave interference leads to increases in signal uncertainty and complexity, making the entropy value [31,32].

### 4.3. Insulin Resistance: Bidirectional Link between MetS and NAFLD

In general, the increased prevalence of MetS is primarily a result of overnutrition and a sedentary lifestyle. MetS is a key risk factor for cardiovascular disease incidence and mortality, as well as for all-cause mortality [46]. The central etiological cause of MetS is commonly considered to be insulin resistance, which is defined as the failure of insulin to stimulate glucose transport to its target cells [47]. Insulin is a pleiotropic hormone that regulates several cell functions, including stimulation of glucose transport, cell growth, energy balance, and regulation of gene expression [48]. The functions of insulin are associated with two signal pathways: the phosphatidylinositol 3-kinase-protein kinase B pathway and mitogen-activated protein kinase pathway [49]. 

Once these signal pathways have been altered, insulin resistance is initialized. Free fatty acids (FFAs) play a key role in the development of insulin resistance [49]. As insulin resistance develops, a large quantity of plasma FFAs are released by white adipose tissues into the liver, leading to hepatic fat accumulation [50]. At the same time, overnutrition and a sedentary lifestyle closely correlate with the occurrence of NAFLD. In those who suffer from NAFLD, hepatic fat accumulation can result in hepatic insulin resistance to strengthen the behavior of MetS [49]. Therefore, insulin resistance could be considered the bidirectional link between MetS and NAFLD [49,51].

### 4.4. Superiority of Entropy in the Assessment of NAFLD and MetS

Several studies have clearly indicated that NAFLD is not only a cause of liver disease but also a key risk indicator of cardiovascular disease [52,53,54]. Patients with both NAFLD and MetS have an increased risk of cardiovascular disease [55]. For these reasons, a quantitative ultrasound parameter used for evaluating NAFLD should satisfy two requirements: (i) changes in liver microstructures during fatty infiltration can be described and explained from a histological viewpoint, and (ii) significant metabolic information can be reflected to satisfy a variety of clinical applications. In this study, both the ASQ and entropy imaging were shown to able to characterize NAFLD and MetS. Compared with the ASQ, however, entropy imaging better fulfills the above two requirements, as supported by the current results. First, the entropy value of ultrasound entropy imaging is more relevant than the FD ratio of the ASQ to the US-FLI (Figure 2), representing that the entropy image characterizes NAFLD more effectively. Second, the entropy value better predicted the risk of MetS than the FD ratio did (Table 3), demonstrating that entropy imaging links metabolic information more strongly.

Possible mechanisms for why ultrasound entropy imaging provides improved performances in evaluating NAFLD and MetS are discussed below. As mentioned in Section 4.3, NAFLD and MetS interact with each other. Consequently, as long as ultrasound parameters can robustly and precisely describe changes in the backscattered statistics during the process of fatty infiltration in the liver, the opportunities to show more metabolic information increase. From this viewpoint, ultrasound entropy imaging is superior to the ASQ technique. As reviewed in the Introduction, the ASQ technique based on the analysis of ultrasound backscattered statistics is gaining attention for the diagnosis of NAFLD. Some animal studies have revealed that ASQ has a high ability to detect hepatic steatosis [20,24,45]; however, its value in quantifying the degree of hepatic steatosis in human liver remains in dispute because of inconsistent findings. For example, Son et al. demonstrated that the FD ratio correlated with the hepatic fat fraction (HFF) measured using MRS (*r* = −0.87; *p* < 0.001) [21], whereas Karlas et al. found that the FD ratio did not significantly correlate with the HFF (*r* = −0.43; *p* = 0.004) [22]. Failure to use the same procedures and settings for ASQ measurements may be one cause for inconsistent findings. The criteria used for rejecting envelope signals and comparing $C_{m}^{2}$ and *r*$C_{m}^{2}$ in the ASQ algorithm may also result in uncertainty in ASQ analysis [33] because these criteria are empirically determined in practical applications [19,45]. By contrast, ultrasound entropy imaging does not require additional signal rejection criteria, and thus it is less influenced by the effects of computational settings and parameter tuning. In addition, the advantages of information entropy lie in entropy estimation no longer being limited by the statistical properties of signals [30,32], implying that entropy is a data-adaptive parameter for ultrasound tissue characterization. A relatively simple but more adaptive computational scheme enables ultrasound entropy imaging to robustly and stably perform NAFLD evaluations, as supported by histopathological validations of both the animal model [32] and clinical trials [31,33]. These reasons explain why ultrasound entropy imaging correlates with MetS more significantly than does ASQ. In other words, when entropy works for characterizing NAFLD, it simultaneously provides significantly metabolic meanings that benefit evaluations in various aspects.

### 4.5. Comparison with Related Studies

A novel parameter named the controlled attenuation parameter (CAP) has been developed based on the properties of ultrasonic signals acquired by transient elastography (Fibroscan^®^). The CAP was demonstrated to correlate with fat accumulation in the liver [56,57] and facilitate the diagnosis of hepatic steatosis [58,59]. Furthermore, one study found that the CAP correlated with several MetS components [60]. However, the question of whether the CAP can perform well in NAFLD diagnosis remains unanswered because unfavorable diagnoses have been reported [61,62,63]. This is likely because the meaning of the CAP corresponds to the viscoelastic properties of the liver but does not provide information on changes in the microstructure, which is crucial in the clinical evaluation of hepatic steatosis. In the future, combining entropy imaging with the CAP may be a feasible strategy for a more complete evaluation of NAFLD and MetS than either one alone.

### 4.6. Limitations of This Study

This study had two limitations. First, the effect of body habitus on the association of entropy and ASQ analysis with MetS was not investigated. For instance, obesity may restrain quantitative measurements of ultrasonography. Second, the original equipment (Toshiba systems and software packages) was unavailable for ASQ analysis. Therefore, we implemented ASQ analysis by using backscattered envelope data acquired from the Terason system. We cannot deny that some bias of estimation accuracy of the FD ratio may have existed among system platforms. However, prior to this study, we fine-tuned the parameters of the algorithm to mitigate this concern.

## 5. Conclusions

In this study, we performed ultrasound entropy imaging of NAFLD and studied its association with MetS through comparisons with biochemical examinations and HOMA-IR (the measure of insulin resistance). In addition, the dependency of entropy imaging on MetS was compared with that of the conventional ASQ technique to explore possible strengths. The clinical results showed that the entropy value was more closely correlated with the US-FLI than the FD ratio of ASQ analysis, indicating that ultrasound entropy imaging improved the performance of NAFLD evaluation. Moreover, the entropy value and FD ratio correlated with metabolic indices and HOMA-IR, thereby confirming metabolic meanings of quantitative ultrasound. Notably, after adjustment for confounding factors, ultrasound entropy still provided a stronger link to the risk of MetS than did the FD ratio according to statistical OR analysis. Compared with model-based ASQ, ultrasound entropy imaging not only characterizes liver microstructures but also reflects metabolic information of NAFLD more significantly. Thus, ultrasound entropy imaging—a data-adaptive approach for tissue characterization—may play a key role in the evaluation of NAFLD and MetS.

## Figures and Tables

**Table 1:** Patient characteristics (n = 394).

Variables	Value *
Questionnaires	
Gender F/M	243/151
Age (yrs)	40.5 ± 11.3 (20–72)
Menopause	25 (6.4)
Smoking	
Never	336 (85.3)
Current	42 (10.7)
Previous	16 (4.1)
Alcohol	
Never	322 (81.7)
Current	64 (16.2)
Previous	8 (2)
Betel Nuts	
Never	375 (95.2)
Current	19 (4.8)
Exercise time (mins/per week)	99.6 ± 189.4 (0–1500)
Anthropometric variable	
BMI (kg/m^2^)	24.1 ± 4.6 (14.8–43.7)
Waist (cm)	81.9 ± 11.3 (55–123)
SBP (mmHg)	122.5 ± 16.3 (86–180)
DBP (mmHg)	77.9 ± 11.9 (50–133)
Biochemistry parameters	
FPG (mg/dL)	87.7 ± 17.6 (58–272)
TCH (mg/dL)	192.9 ± 35.5 (101–320)
TG (mg/dL)	112.4 ± 90.3 (25–888)
HDL-C (mg/dL)	57.3 ± 15.8 (25–120)
LDL-C (mg/dL)	120.8 ± 32.5 (47–238)
AST (U/L)	22.9 ± 8.9 (11–68)
ALT (U/L)	26.5 ± 21.4 (2–151)
Insulin (μU/mL)	9.1 ± 8.2 (2–84.4)
HOMA-IR	1.17 ± 1.03 (0.26–10.2)
MetS (%)	76 (19.3)
Ultrasound parameters	
US-FLI Score	2.22 ± 2.25 (0–8)
ASQ FD-ratio	0.96 ± 0.44 (0.21–2.89)
Entropy	3.99 ± 0.06 (3.80–4.07)

* Categorical data are expressed as numbers (percentage); continuous variables are expressed as mean ± SD (range). BMI: body mass index; SBP: systolic blood pressure; DBP: diastolic blood pressure; FPG: fasting plasma glucose; TCH: total cholesterol; TG: triglycerides; HDL-C: high-density lipoprotein cholesterol; LDL-C: low-density lipoprotein cholesterol; AST: aspartate aminotransferase; ALT: alanine aminotransferase; HOMA-IR: homeostasis model assessment for insulin resistance; MetS: metabolic syndrome.

**Table 2:** Characteristics of participants in different tertiles of ultrasound quantitative parameters.

	Entropy				ASQ FD-ratio			
Variables	1_st_ tertile	2_nd_ tertile	3_rd_ tertile	*p*-value	1_st_ tertile	2_nd_ tertile	3_rd_ tertile	*p*-value
No. of participants	131	131	132		131	131	132	
Gender F/M	113/18	76/55	54/78	<0.0001	65/66	77/54	101/31	<0.0001
Age (yrs)	38.33 ± 9.87	41.6 ± 11.86	41.7 ± 11.81	0.009	41.40 ± 11.79	40.79 ± 11.02	39.40 ± 11.07	0.2602
Waist (cm)	73.31 ± 7.28	81.96 ± 9.9	90.43 ± 9.18	<0.0001	88.24 ± 10.98	82.61 ± 9.99	74.79 ± 8.32	<0.0001
BMI * (kg/m^2^)	20.83 ± 2.35	24.2 ± 4.05	27.29 ± 4.41	<0.0001	26.35 ± 4.47	24.61 ± 4.70	21.40 ± 2.78	<0.0001
SBP (mmHg)	114.88 ± 13.1	121.98 ± 16.97	130.58 ± 14.81	<0.0001	128.76 ± 16.74	122.99 ± 15.48	115.76 ± 14.06	<0.0001
DBP (mmHg)	73.58 ± 9.93	76.81 ± 12.44	83.36 ± 11.27	<0.0001	81.56 ± 11.95	77.82 ± 12.00	74.42 ± 10.86	<0.0001
FPG (mg/dL)	81.64 ± 8.36	86.95 ± 11.45	94.47 ± 25.39	<0.0001	92.85 ± 24.77	87.59 ± 13.60	82.70 ± 9.13	<0.0001
TCH (mg/dL)	181.29 ± 31.12	194.95 ± 35.77	202.33 ± 36.34	<0.0001	198.20 ± 36.30	195.49 ± 36.13	185.01 ± 32.87	0.0353
TG (mg/dL)	65.44 ± 28.39	106.46 ± 67.7	164.86 ± 118.71	<0.0001	151.11 ± 121.47	111.71 ± 72.79	74.63 ± 40.21	<0.0001
HDL-C (mg/dL)	65.66 ± 13.78	57.03 ± 14.59	49.17 ± 14.63	<0.0001	50.66 ± 12.33	56.26 ± 17.57	64.82 ± 13.83	<0.0001
LDL-C (mg/dL)	106.73 ± 27.43	123.6 ± 31.9	131.98 ± 32.79	<0.0001	127.57 ± 32.65	124.27 ± 31.24	110.64 ± 31.23	0.0022
AST (U/L)	19.60 ± 5.92	22.59 ± 8.48	26.37 ± 10.42	<0.0001	26.68 ± 10.72	22.49 ± 8.50	19.45 ± 5.06	<0.0001
ALT (U/L)	15.84 ± 7.53	25.01 ± 18.65	38.67 ± 26.67	<0.0001	39.51 ± 28.02	24.93 ± 16.31	15.26 ± 5.99	<0.0001
Insulin (μU/mL)	5.52 ± 3.4	9.06 ± 9.29	12.05 ± 8.7	<0.0001	10.71 ± 7.54	10.44 ± 10.63	5.7 ± 3.49	<0.0001
HOMA-IR	0.71 ± 0.44	1.16 ± 1.14	1.56 ± 1.10	<0.0001	1.39 ± 0.98	1.33 ± 1.31	0.73 ± 0.45	<0.0001
MetS (%)	1 (0.8%)	20 (15.3%)	55 (41.7%)	<0.0001	44 (33.6%)	24 (18.3%)	8 (6.1%)	<0.0001
US-FLI Score	0.53 ± 0.9	1.71 ± 1.55	4.42 ± 2.0	<0.0001	3.82 ± 2.40	2.18 ± 1.82	0.67 ± 1.09	<0.0001

* BMI: body mass index; SBP: systolic blood pressure; DBP: diastolic blood pressure; FPG: fasting plasma glucose; TCH: total cholesterol; TG: triglycerides; HDL-C: high-density lipoprotein cholesterol; LDL-C: low-density lipoprotein cholesterol; AST: aspartate aminotransferase; ALT: alanine aminotransferase; HOMA-IR: homeostasis model assessment for insulin resistance; MetS: metabolic syndrome.

**Table 3:** ORs in each tertile of entropy and the FD (focal disturbance) ratio for evaluating the risk of MetS.

	Entropy				ASQ FD-ratio			
	1_st_ tertile	2_nd_ tertile	3_rd_ tertile	*p*-value	1_st_ tertile	2_nd_ tertile	3_rd_ tertile	*p*-value
	(n = 131)	(n = 131)	(n = 132)		(n = 131)	(n = 131)	(n = 132)	
Model 1 *	ref	51.29 (2.76–164.43)	85.57 (11.25–650.56)	<0.0001	ref	0.48 (0.26–0.89)	0.04 (0.01–0.14)	<0.0001
Model 2	ref	10.27 (1.29–82.14)	26.84 (3.34–215.4)	0.0007	ref	0.59 (0.30–1.18)	0.41 (0.16–1.05)	0.1144
Model 3	ref	7.91 (0.96–65.18)	20.47 (2.48–168.67)	0.0021	ref	0.55 (0.27–1.14)	0.42 (0.15–1.17)	0.13

* Model 1: adjusted for age, gender, smoking, alcohol consumption, betel nut chewing, hours of exercise per week, and menopause status (women only). Model 2: same as model 1 plus further adjustment for BMI. Model 3: model 2 plus further adjustment for HOMA-IR.
